# Exercise Endocrinology: Guidance for Future Research Direction and Focus

**DOI:** 10.4172/2157-7536.1000.e114

**Published:** 2015-01-12

**Authors:** Anthony C Hackney, Amy R Lane

**Affiliations:** 1 Human Movement Science Curriculum, University of North Carolina - Chapel Hill, NC, USA; 2 Department of Exercise & Sport Science, University of North Carolina - Chapel Hill, NC, USA; 3 Department of Nutrition - Gillings School of Global Public Health, University of North Carolina - Chapel Hill, NC, USA

## Editorial

Physical activity and exercise are behavioral components that the major world health organizations (e.g. CDC, WHO) have necessitated as essential for all individuals to incorporate into their lifestyle in order to be healthy and more productive. To this end, an increasing number of individuals world-wide are attempting to engage in exercise training on a regular basis to reap these well-being benefits. The physiological adaptations occurring in response to exercise training are well documented and come about due to the plasticity of the tissues and organs of the human body [1]. Enhanced energy metabolism, cardiovascular function, hematological parameters, muscular strength-endurance and many more adaptations occur to facilitate improved exercise and physical work capacity following training. A key and integral physiologic component in enabling and regulating this adaptation process is the endocrine system and the hormone-like substances associated with the system [2,3].

The study of Exercise Endocrinology began in the late 1960s, shortly after the advancement of the radioimmunoassay (RIA) procedural technique that allowed for more accurate assessment of hormonal substances in humans and animals [4]. Arguably one of the most prominent early researchers in this field was Dr. Atko Viru of Tartu University in Estonia (through most of his career, part of the Soviet Union). Dr. Viru's work (over 500 research publications) led to groundbreaking discoveries about the responses of hormones to acute and chronic exercise exposure, as well as to the mechanisms of why the responses were occurring in the physiological “internal milieu”. As a young researcher I (ACH) was fortunate to work in Dr. Viru's laboratory and study under his direction. Regrettably he is no longer with us; but some of the questions he began studying decades ago are still in need of further addressing and attention within the specialized field of exercise endocrinology. Put simply, the approach to studying hormones and exercise he developed can be viewed as attempting to address three basic questions; 1) what happens? - How do hormones change in response to an exercise session, 2) why does it happen? -What is the physiological role of the hormonal response to exercise, and 3) how does it happen? - Mechanistically, what is the means for regulating and inducing the responses.

In our opinion, unfortunately, many young exercise physiologists and endocrinologist researchers seem to be focusing their research agendas on the first of his questions extensively. While “what happens?” is a viable question, it is a question that has been extensively addressed and well defined for many of the existing known hormonelike substances. In other words some researchers are addressing a question that has already been well answered. This approach of repeatedly answering the same question provides for duplication and redundancy of information in the field. It is also “safe science” as it allows for researchers to have high levels of internal-external validity to their findings - i.e., agreeing with what is already known. There is unquestionably a need in science for replication of studies to substantiate the robustness of previous findings; but, when carried to excess it undermines one of the key principles of the Scientific Method - “acquiring new knowledge”.

To this end, there is a strong need for researchers to readjust their focus and research aims on more aspects of questions two and three noted above. These are the areas where far fewer studies have been conducted and many unknowns exist within exercise endocrinology relative to the questions. These questions, however, are more difficult to address in research designs and require more advanced technical procedures and approaches within methodologies. Nonetheless, research studies incorporating and approaching aspects of questions two and three will provide much needed insight into the understanding of the means by which the endocrine system enables and regulates the adaptation process with respect to exercise and exercise training. This is especially true with responses related to molecular endocrinology and the epigenetic influences of exercise on the endocrine system. New research along these lines will provide greater insight into how exercise can be used more effectively as a means to modify disease states and their comorbidities. That is, provide more understanding as to how “Exercise is Medicine”^✉^ in this modern age [5].

We want to recommend and encourage researchers in exercise endocrinology, as they look to the future and develop new research projects that they focus more of their attention on addressing the second and third questions proposed by Dr. Viru - Why does it happen? - What is the physiological role of the hormonal response to exercise?; and, How does it happen? - Mechanistically what is the means for regulating and inducing the responses. These are challenging questions, but the answers will be exciting, stimulating and insightful as to how the exercising human body works and adapts.
